# Anti-synthetase syndrome is associated with a higher risk of hospitalization among patients with idiopathic inflammatory myopathy and COVID-19

**DOI:** 10.3389/fimmu.2024.1295472

**Published:** 2024-03-04

**Authors:** Wanlong Wu, Runci Wang, Cuiying Xie, Yi Chen, Xiangyu Teng, Shuhui Sun, Wenwen Xu, Yakai Fu, Yiyangzi Ma, Antao Xu, Xia Lyu, Yan Ye, Jia Li, Chunyan Zhang, Nan Shen, Xiaodong Wang, Shuang Ye, Qiong Fu

**Affiliations:** ^1^ Department of Rheumatology, Renji Hospital, Shanghai Jiao Tong University School of Medicine, Shanghai, China; ^2^ Department of Emergency Medicine, Renji Hospital, Shanghai Jiao Tong University School of Medicine, Shanghai, China

**Keywords:** COVID-19, idiopathic inflammatory myopathy, interstitial lung disease, anti-synthetase syndrome, JAK inhibitor

## Abstract

**Background:**

Data with fine granularity about COVID-19-related outcomes and risk factors were still limited in the idiopathic inflammatory myopathies (IIMs) population. This study aimed to investigate clinical factors associated with hospitalized and severe COVID-19 in patients with IIMs, particularly those gauged by myositis-specific antibodies.

**Methods:**

This retrospective cohort study was conducted in the Renji IIM cohort in Shanghai, China, under an upsurge of SARS-CoV-2 omicron variant infections from December 2022 to January 2023. Clinical data were collected and analyzed by multivariable logistic regression to determine risk factors. High-dimensional flow cytometry analysis was performed to outline the immunological features.

**Results:**

Among 463 infected patients in the eligible cohort (n=613), 65 (14.0%) were hospitalized, 19 (4.1%) suffered severe COVID-19, and 10 (2.2%) died. Older age (OR=1.59/decade, 95% CI 1.18 to 2.16, p=0.003), requiring family oxygen supplement (2.62, 1.11 to 6.19, 0.028), patients with anti-synthetase syndrome (ASyS) (2.88, 1.12 to 7.34, 0.027, vs. other dermatomyositis), higher IIM disease activity, and prednisone intake >10mg/day (5.59, 2.70 to 11.57, <0.001) were associated with a higher risk of hospitalization. Conversely, 3-dose inactivated vaccination reduced the risk of hospitalization (0.10, 0.02 to 0.40, 0.001, vs. incomplete vaccination). Janus kinase inhibitor (JAKi) pre-exposure significantly reduced the risk of severe COVID-19 in hospitalized patients (0.16, 0.04 to 0.74, 0.019, vs. csDMARDs). ASyS patients with severe COVID-19 had significantly reduced peripheral CD4+ T cells, lower CD4/CD8 ratio, and fewer naive B cells but more class-switched memory B cells compared with controls.

**Conclusion:**

ASyS and family oxygen supplement were first identified as risk factors for COVID-19-related hospitalization in patients with IIMs. JAKi pre-exposure might protect IIM patients against severe COVID-19 complications.

## Introduction

The omicron variant of severe acute respiratory syndrome coronavirus-2 (SARS-CoV-2), BA.5.2, has stroked China mainland in Dec. 2022 after a national open-up policy change. Within 2 months, an upsurge of excess hospitalization and deaths was observed. In the current study, we investigated a special cohort of patients with idiopathic inflammatory myopathies (IIMs) during this critical period.

IIMs are a heterogeneous group of autoimmune disorders, characterized by varying degrees of cutaneous lesions, myositis, and frequent pulmonary involvement (1). Patients with IIM are considered vulnerable to Coronavirus Disease 2019 (COVID-19) due to the underlying immune disturbance and a high prevalence of interstitial lung disease (ILD) (2). In general, it has been established from global registry and cohort studies on rheumatic diseases that older age, male sex, more comorbidities, higher disease activity, exposure to glucocorticoid (GC) (prednisone ≥10 mg/day), and certain disease-modifying antirheumatic drugs (DMARDs) (e.g., rituximab) are associated with severe COVID-19 and unfavorable outcomes (3–6). Other synthetic or biological DMARDs were not associated with an increased risk of severe COVID-19 and COVID-19-related death in previous literature (4–6). Conversely, sufficient vaccination and timely outpatient anti-viral therapy might reduce the risk of COVID-19-related hospitalization or death in patients with rheumatic conditions (7–9).

However, data about COVID-19-related outcomes and risk factors were still short of supply in the well-defined IIM population (10, 11). In particular, certain myositis-specific antibodies (MSAs)-gauged subtypes of IIM, e.g., anti-synthetase syndrome (ASyS) and anti-melanoma differentiation-associated gene 5 (MDA5) positive dermatomyositis (DM), are more frequently complicated with significant ILD (12, 13). The clinical features as well as immunophenotyping of these patients superimposed with COVID-19 were largely unknown.

Thus, we conducted a retrospective study in an established IIM cohort from a single tertiary referral center at Renji Hospital in Shanghai, China, to investigate clinical factors associated with hospitalized and severe COVID-19, and outline immunological features in IIM patients with COVID-19.

## Methods

### Patients and outcome measures

Eligible patients needed to meet the following inclusion criteria: IIM diagnosis fulfilling 2017 EULAR/ACR classification criteria or 239th European Neuromuscular Centre dermatomyositis criteria (14, 15), definite MSAs result, definite data for SARS-CoV-2 infection by the index date (20 January 2023), and related outcome data, i.e., hospitalization, severity of COVID-19, or death.

SARS-CoV-2 infection was pathogen-confirmed by positive PCR or antigen test. Severe COVID-19 was defined as new-onset or deteriorating respiratory failure requiring high-flow oxygen support or beyond, including noninvasive ventilation, invasive ventilation, or extracorporeal membrane oxygenation (16). Hospitalized patients not satisfying the above severe criteria were defined as moderate COVID-19.

Written informed consent was obtained from each participating patient or designated surrogate. Clinical data and peripheral blood samples were collected under the study protocol approved by the Ethics Committee of Renji Hospital (identification no. 2013-126, RA-2023-101).

### Demographic and clinical data collection

Demographic data (age, sex), smoking status, body mass index, disease characteristics (MSAs, disease duration, IIM-associated ILD, oxygen supplement status prior to infection, IIM disease activity, and comorbidities), immunosuppressive medications for IIM, vaccination data prior to COVID-19 or the index date, negative conversion time of antigen test, and length of hospitalization were collected for analyses.

MSAs were identified by commercial immunoblotting assay with EUROLINE Autoimmune Inflammatory Myopathies 16 Ag (IgG) (Euroimmun, Lubeck, Germany). Patients were categorized into four subgroups according to MSAs: anti-MDA5 positive DM, ASyS (i.e., anti-Jo1, anti-EJ, anti-PL7, anti-PL12, anti-OJ positive), immune-mediated necrotizing myopathy (IMNM) (i.e., anti-SRP, anti-HMGCR positive), and other DM (i.e., anti-TIF1γ, anti-NXP2, anti-Mi2, anti-SAE positive, or MSA negative but fulfill the aforementioned DM criteria).

IIM disease status was assessed by the patient’s global assessment (PtGA), which was categorized as remission, low, moderate or high disease activities, respectively.

Comorbidities including hypertension, coronary heart disease, diabetes, malignancy, chronic obstructive pulmonary disease, chronic renal disease, and stroke were recorded. The number of comorbidities rather than specific disorders was used for analyses.

Immunosuppressive medications for IIM prior to COVID-19 or the index date were recorded, which included prednisone-equivalent GC dose, and immunosuppressants (IS). Prednisone dose was dichotomized to ≤10mg per day and >10mg per day. IS were categorized as follows for statistics: conventional synthetic disease-modifying antirheumatic drugs (csDMARDs) including methotrexate, azathioprine, leflunomide, cyclosporine, tacrolimus, mycophenolate mofetil, cyclophosphamide (within 6 months), and iguratimod; biological DMARDs (bDMARDs) including rituximab (within 6 month) and tocilizumab (within 1 month); Janus kinase inhibitors (JAKi), i.e., tofacitinib and baricitinib. When csDMARDs were prescribed together with bDMARDs or JAKi in one patient, this subject was regarded as using bDMARDs or JAKi in the statistical analysis. Finally, the number of IS (i.e., none, mono, combined) was also counted.

COVID-19 vaccination data (all inactivated vaccine) were summarized into three groups as follows: incomplete (0-1 dose), full (2 doses), and booster (3 doses or more).

### Immunophenotype analysis

Up till now, the immunological features of IIM patients during COVID-19 infection remain unknown. In search of distinct immunological features as potential biomarkers to promptly identify high-risk patients, we performed flow cytometry analysis of peripheral blood mononuclear cells (PBMC) from IIM+COVID-19 donors and COVID-19 control donors. Blood samples were collected in hospitalized IIM patients. Hospitalized COVID-19 patients without rheumatic diseases were recruited as controls. PBMCs were isolated and acquired on a BD Fortessa analyzer using FACSDiva software. Data were analyzed using FlowJo 10.8.1. The gating strategy used in flow cytometry manual gating is shown in **Supplementary Figure S1**.

For high-dimensional analysis, fcs files from individual samples were labeled with sample ID, condition, disease severity, and clinical outcome and concatenated into one fcs file. In total, 221,696 viable T cells from 35 samples were used for downstream analysis. Dimensional reduction was performed using opt-SNE from 1000 iterations with a perplexity = 30. Hierarchical consensus clustering was performed on CD4+ and CD8+ T cells respectively using the FlowSOM algorithm to generate 10 metaclusters (populations) from 100 iterations. Thirteen clustering channels excluding gating markers were used for analyses. A heatmap of row-normalized median expression of clustering markers in the metaclusters was shown, in which the metaclusters and markers were arranged by hierarchical consensus clustering. Markers of interest were overlaid on the tSNE plot for visualization. Manual biaxial gating was performed on individual fcs files and the concatenated files using FlowJo v.10.8.1 for quality control and independent examination of the expression of markers and frequencies of populations.

### Statistical analysis

Continuous variables were summarized as mean (SD) while categorical variables were summarized as number (percentage). Patient’s clinical data were compared between patients with and without COVID-19, requiring and not-requiring COVID-19-related hospitalization, with severe and non-severe COVID-19, respectively, by independent sample *t*-test, Mann–Whitney U-test, chi-square test, and Fisher’s exact test, as appropriate.

The primary analysis was performed to determine the factors associated with COVID-19-related hospitalization in infected IIM patients by multivariable logistic regression. A secondary multivariable logistic regression was conducted in the COVID-19-related hospitalized IIM population to determine the factors associated with severe COVID-19.

In the high-dimensional flow cytometry analysis, altered metaclusters were identified by comparing the abundance of each metaclusters among condition, severity, or outcome groups using two-way ANOVA. Altered frequencies of manually gated populations were identified by comparison among condition, severity, or outcome groups using the Kruskal-Wallis test with Dunn’s multiple comparison correction.

All candidate predictors with *p*<0.1 in univariable analysis and important confounders with clinical significance were incorporated in the multivariable logistic regression (17–19). No imputation method was conducted since no data was missing in the listed variables. The backward (LR) method was applied to select the best logistic regression model to avoid overfitting. All aforementioned statistical procedures were performed by Prism 9 and R V.4.2.2 software. Significance was defined as *p <*0.05.

## Results

### Study population

**Figure 1:**
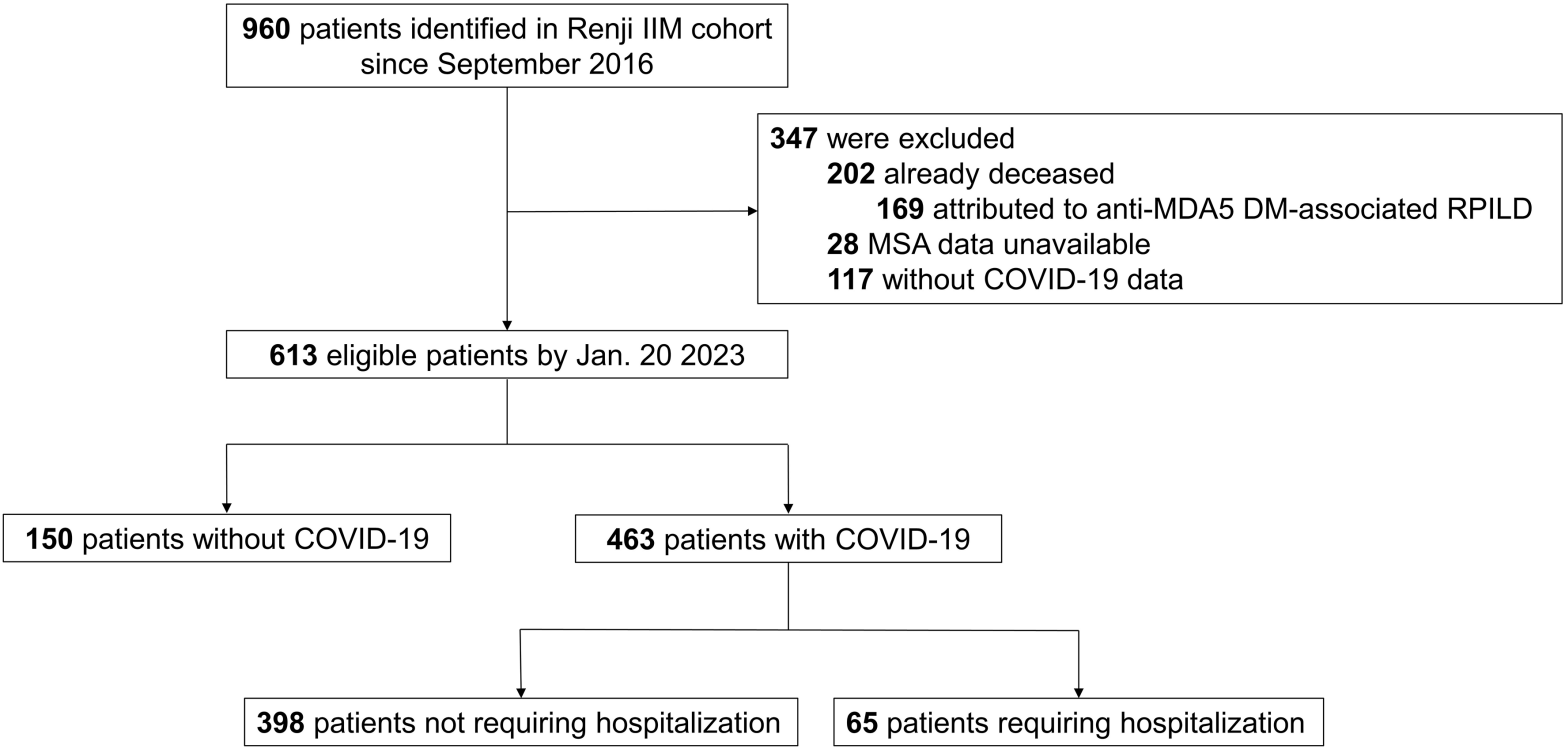
Study population. IIM, idiopathic inflammatory myopathy; MDA5, melanoma differentiation-associated gene 5; DM, dermatomyositis; RPILD, rapid-progressive interstitial lung disease; MSA; myositis-specific antibody; COVID-19, Coronavirus Disease 2019.

A total of 960 patients in the Renji IIM cohort from September 2016 were identified. By 20 January 2023 (the index date), 202 patients had already died (of these, 169 deaths were attributed to rapid progressive ILD secondary to anti-MDA5 DM). The MSA data for 28 patients were unavailable. Another 117 patients did not have definite data for SARS-CoV-2 infection. As a result, complete COVID-19 and related outcome data were available for 613 subjects for subsequent analyses (**Figure 1**).

Patients were predominantly female (73.2%) with a mean age of 52.2 (12.9) years. The mean disease duration was 3.6 (2.9) years. The majority (84.3%) had IIM-associated ILD, while 60 patients (9.8%) required family oxygen supplement. A total of 371 (60.5%) patients did not have any recorded comorbidity, while 171 (27.9%) had one comorbidity, and 71 (11.6%) had two or more comorbidities. A minority of patients were ever (9.6%) or current (3.8%) smokers. Only 30.8% of patients were fully vaccinated, i.e., 107 (17.5%) patients had two doses and 82 (13.4%) patients had 3 doses or more. Almost all vaccinations were conducted more than half a year before the index date. The subgroups were dominated by anti-MDA5 DM (44.7%), followed by ASyS (31.0%), other DM (19.6%, which included 4.4% of those with MSA-negative DM), and IMNM (4.7%). A total of 235 (38.3%) patients had disease remission prior to COVID-19 or the index date according to patient’s global assessment, while 260 (42.4%), 100 (16.3%), and 18 (2.9%) patients had low, moderate, and high disease activities, respectively. The mean prednisone-equivalent GC dose was 10.2 (10.7) mg per day. Among those, 177 (28.9%) patients received GC with a dose of more than 10mg per day, while GC had been discontinued in 73 (11.9%) patients. Up to 447 (72.9%) patients received one IS; while 60 (9.8%) subjects received combined IS, which were exclusively dual-combo IS. JAKi with or without csDMARDs (52.9%) was the most frequently used regimen, followed by sole csDMARDs (23.2%), rituximab with or without csDMARDs (5.7%) and tocilizumab with or without csDMARDs (1.0%) (**Table 1**).

**Table 1:** Univariable comparisons of clinical features prior to COVID-19 or the index date between IIM patients with and without COVID-19.

	Overall (n=613)	No infection (n=150)	Infection (n=463)	p value
Age, years	52.2 (12.9)	52.5 (13.3)	52.1 (12.8)	0.734
Sex				0.729
Female	449 (73.2%)	112 (74.7%)	337 (72.8%)	
Male	164 (26.8%)	38 (25.3%)	126 (27.2%)	
Disease duration, years	3.6 (2.9)	3.4 (2.8)	3.6 (3.0)	0.402
Interstitial lung disease	517 (84.3%)	124 (82.7%)	393 (84.9%)	0.604
Family oxygen supplement	60 (9.8%)	14 (9.3%)	46 (9.9%)	0.954
MSAs-gauged IIM subtypes				0.560
Anti-MDA5 DM	274 (44.7%)	73 (48.7%)	201 (43.4%)	
ASyS	190 (31.0%)	40 (26.7%)	150 (32.4%)	
IMNM	29 (4.7%)	8 (5.3%)	21 (4.5%)	
Other DM	120 (19.6%)	29 (19.3%)	91 (19.7%)	
Vaccination				0.419
Incomplete (0-1 dose)	424 (69.2%)	107 (71.3%)	317 (68.5%)	
Full (2 doses)	107 (17.5%)	21 (14.0%)	86 (18.6%)	
Booster (3 doses or more)	82 (13.4%)	22 (14.7%)	60 (13.0%)	
IIM disease activity (PtGA)				0.399
Remission	235 (38.3%)	53 (35.3%)	182 (39.3%)	
Low	260 (42.4%)	68 (45.3%)	192 (41.5%)	
Moderate	100 (16.3%)	27 (18.0%)	73 (15.8%)	
High	18 (2.9%)	2 (1.3%)	16 (3.5%)	
Prednisone, mg/day	10.2 (10.7)	10.3 (10.3)	10.2 (10.8)	0.897
Prednisone dose>10mg/day	177 (28.9%)	48 (32.0%)	129 (27.9%)	0.385
Withdrawn of GC	73 (11.9)	21 (14.0)	52 (11.2)	0.444
No. of immunosuppressant				0.244
None	106 (17.3%)	30 (20.0%)	76 (16.4%)	
Mono	447 (72.9%)	110 (73.3%)	337 (72.8%)	
Combined	60 (9.8%)	10 (6.7%)	50 (10.8%)	
Type of immunosuppressant				0.054
None	106 (17.3%)	30 (20.0%)	76 (16.4%)	
JAK inhibitor	324 (52.9%)	83 (55.3%)	241 (52.1%)	
Sole csDMARDs	142 (23.2%)	27 (18.0%)	115 (24.8%)	
Rituximab	35 (5.7%)	6 (4.0%)	29 (6.3%)	
Tocilizumab	6 (1.0%)	4 (2.7%)	2 (0.4%)	
No. of comorbidity				0.973
None	371 (60.5%)	92 (61.3%)	279 (60.3%)	
One	171 (27.9%)	41 (27.3%)	130 (28.1%)	
Two or more	71 (11.6%)	17 (11.3%)	54 (11.7%)	
Smoker				0.791
Never	531 (86.6%)	129 (86.0%)	402 (86.8%)	
Ever	59 (9.6%)	14 (9.3%)	45 (9.7%)	
Current	23 (3.8%)	7 (4.7%)	16 (3.5%)	
BMI, kg/m^2^	23.7 (3.6)	23.3 (3.7)	23.8 (3.6)	0.178
BMI>28	69 (11.3%)	17 (11.3%)	52 (11.2%)	0.973

Data are presented as mean (SD), or n (%).

COVID-19, Coronavirus Disease 2019; IIM, idiopathic inflammatory myopathy; MSA, myositis-specific antibody; MDA5, melanoma differentiation-associated gene 5; DM, dermatomyositis; ASyS, anti-synthetase syndrome; IMNM, immune-mediated necrotizing myopathy; PtGA, patient’s global assessment; GC, glucocorticoid; JAK, Janus kinase; csDMARDs, conventional synthetic disease-modifying antirheumatic drugs; BMI, Body Mass Index.

### COVID-19-related clinical outcome

A total of 463 patients had SARS-CoV-2 infection from 1 December 2022 to 20 January 2023, which yielded a crude 75.5% infection rate in our IIM cohort during this period. The patients’ clinical characteristics and treatment data were similar between patients with and without COVID-19 (**Table 1**). Among the patients infected with SARS-CoV-2, 65 (14.0%) patients needed hospitalization due to COVID-19. Nineteen (4.1%) patients suffered severe COVID-19. Ten (2.2%) patients had died by 28 February 2023.

Compared to those not requiring hospitalization (n=398), hospitalized patients (n=65) were significantly older (59.2 vs. 51.0 years, p<0.001), were more likely to have IIM-associated ILD (93.8% vs. 83.4%, p=0.047), and required more family oxygen supplement (29.2% vs. 6.8%, p<0.001). Patients with COVID-19-related hospitalization were more likely to have ASyS, higher disease activity, and more comorbidities (all p<0.001), yet were less likely to be fully vaccinated (p=0.018). Patients requiring hospitalization received a higher dose of prednisone (19.3 vs. 8.7 mg per day, p<0.001) with a higher percentage of prednisone dose >10mg/day (66.2% vs. 21.6%, p<0.001) and a lower percentage of GC cessation (1.5% vs. 12.8%, p=0.014). In addition, they were more likely to receive combined IS (p=0.010) in the univariable analysis (**Table 2**).

**Table 2:** Univariable comparisons of clinical features prior to COVID-19 and outcomes between infected IIM patients requiring and not requiring COVID-19-related hospitalization.

	Overall (n=463)	No hospitalization (n=398)	Hospitalization (n=65)	p value
Age, years	52.1 (12.8)	51.0 (12.9)	59.2 (9.8)	**<0.001**
Sex				0.081
Female	337 (72.8%)	296 (74.4%)	41 (63.1%)	
Male	126 (27.2%)	102 (25.6%)	24 (36.9%)	
Disease duration, years	3.6 (3.0)	3.6 (2.8)	3.7 (3.8)	0.845
Interstitial lung disease	393 (84.9%)	332 (83.4%)	61 (93.8%)	**0.047**
Family oxygen supplement	46 (9.9%)	27 (6.8%)	19 (29.2%)	**<0.001**
MSAs-gauged IIM subtypes				**<0.001**
Anti-MDA5 DM	201 (43.4%)	187 (47.0%)	14 (21.5%)	
ASyS	150 (32.4%)	109 (27.4%)	41 (63.1%)	
IMNM	21 (4.5%)	20 (5.0%)	1 (1.5%)	
Other DM	91 (19.7%)	82 (20.6%)	9 (13.8%)	
Vaccination				**0.018**
Incomplete (0-1 dose)	317 (68.5%)	263 (66.1%)	54 (83.1%)	
Full (2 doses)	86 (18.6%)	78 (19.6%)	8 (12.3%)	
Booster (3 doses or more)	60 (13.0%)	57 (14.3%)	3 (4.6%)	
IIM disease activity (PtGA)				**<0.001**
Remission	182 (39.3%)	173 (43.5%)	9 (13.8%)	
Low	192 (41.5%)	167 (42.0%)	25 (38.5%)	
Moderate	73 (15.8%)	50 (12.6%)	23 (35.4%)	
High	16 (3.5%)	8 (2.0%)	8 (12.3%)	
Prednisone, mg/day	10.2 (10.8)	8.7 (8.8)	19.3 (16.4)	**<0.001**
Prednisone dose>10mg per day	129 (27.9%)	86 (21.6%)	43 (66.2%)	**<0.001**
Withdrawn of GC	52 (11.2%)	51 (12.8%)	1 (1.5%)	**0.014**
No. of immunosuppressant				**0.010**
None	76 (16.4%)	68 (17.1%)	8 (12.3%)	
Mono	337 (72.8%)	294 (73.9%)	43 (66.2%)	
Combined	50 (10.8%)	36 (9.0%)	14 (21.5%)	
Type of immunosuppressant				0.352
None	76 (16.4%)	68 (17.1%)	8 (12.3%)	
JAK inhibitor	241 (52.1%)	210 (52.8%)	31 (47.7%)	
Sole csDMARDs	115 (24.8%)	96 (24.1%)	19 (29.2%)	
Rituximab	29 (6.3%)	22 (5.5%)	7 (10.8%)	
Tocilizumab	2 (0.4%)	2 (0.5%)	0 (0.0%)	
No. of comorbidity				**<0.001**
None	279 (60.3%)	254 (63.8%)	25 (38.5%)	
One	130 (28.1%)	107 (26.9%)	23 (35.4%)	
Two or more	54 (11.7%)	37 (9.3%)	17 (26.2%)	
Smoker				0.394
Never	402 (86.8%)	349 (87.7%)	53 (81.5%)	
Ever	45 (9.7%)	36 (9.0%)	9 (13.8%)	
Current	16 (3.5%)	13 (3.3%)	3 (4.6%)	
BMI, kg/m^2^	23.8 (3.6)	23.7 (3.7)	24.3 (3.1)	0.253
BMI>28	52 (11.2%)	44 (11.1%)	8 (12.3%)	0.767
Severe COVID-19	19 (4.1%)	0 (0.0%)	19 (29.2%)	**<0.001**
Death	10 (2.2%)	0 (0.0%)	10 (15.4%)	**<0.001**

Data are presented as mean (SD), or n (%). COVID-19, Coronavirus Disease 2019; IIM, idiopathic inflammatory myopathy; MSA, myositis-specific antibody; MDA5; melanoma differentiation-associated gene 5; DM, dermatomyositis; ASyS, anti-synthetase syndrome; IMNM, immune-mediated necrotizing myopathy; PtGA, patient’s global assessment; GC, glucocorticoid; JAK, Janus kinase; csDMARDs, conventional synthetic disease-modifying antirheumatic drugs; BMI, Body Mass Index. P-values<0.05 are highlighted in bold.

### Clinical factors associated with COVID-19-related hospitalization

**Figure 2:**
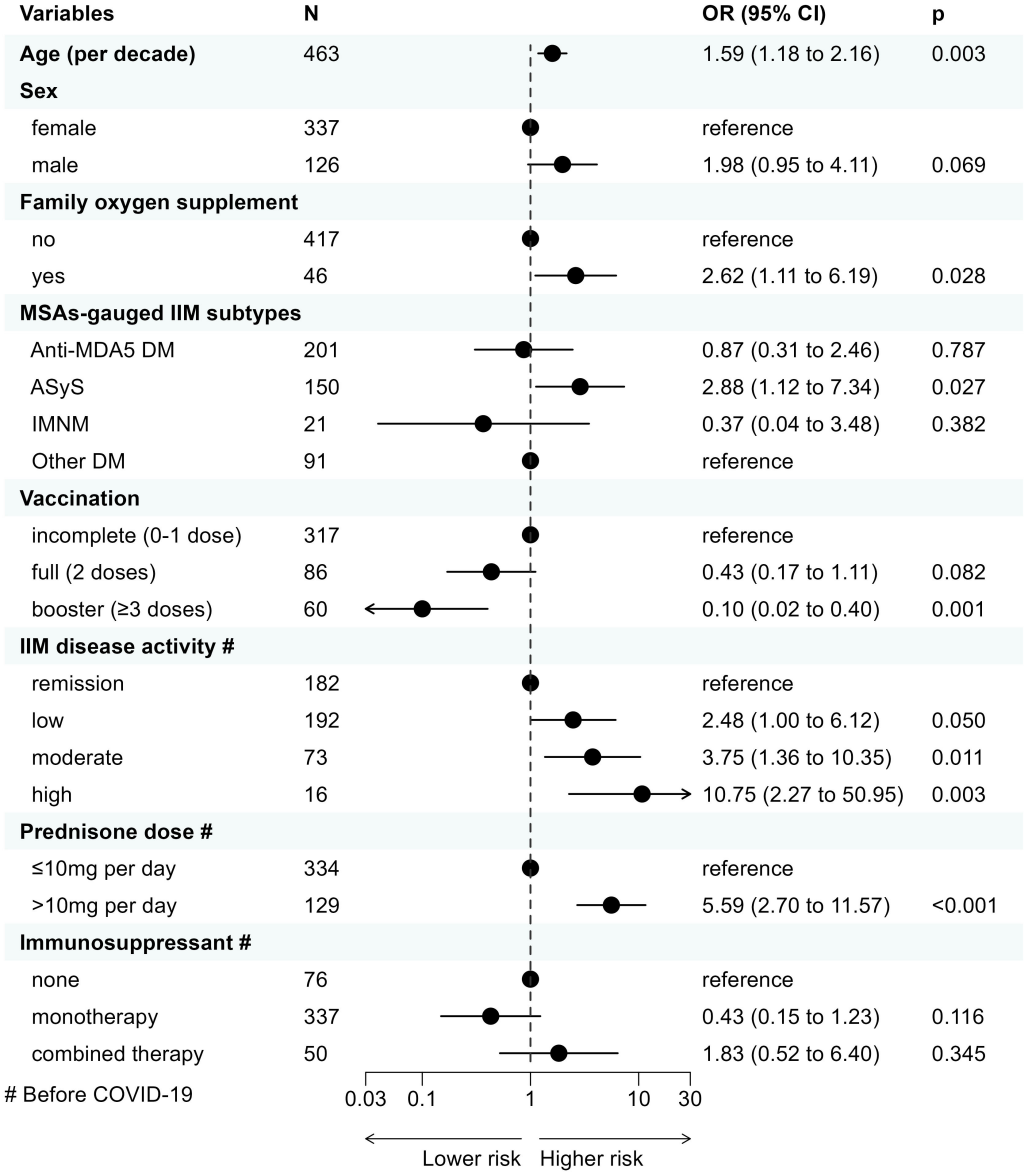
Clinical factors associated with COVID-19-related hospitalization in IIM patients with COVID-19 determined by multivariable logistic regression. COVID-19, Coronavirus Disease 2019; IIM, idiopathic inflammatory myopathy; OR, odds ratio; CI, confidence interval; MSA, myositis-specific antibody; MDA5, melanoma differentiation-associated gene 5; DM, dermatomyositis; ASyS, anti-synthetase syndrome; IMNM, immune-mediated necrotizing myopathy.

In the primary multivariable logistic regression (n=463), older age (OR=1.59 for each additional decade of life, 95% CI 1.18 to 2.16, p=0.003), requiring family oxygen supplement (2.62, 1.11 to 6.19, 0.028, vs. none), ASyS (2.88, 1.12 to 7.34, 0.027; vs. other DM), presence of higher IIM disease activity (2.48 for low disease activity, 1.00 to 6.12, 0.050; 3.75 for moderate disease activity, 1.36 to 10.35, 0.011; 10.75 for high disease activity, 2.27 to 50.95, 0.003; vs. remission, respectively), and GC intake >10mg/day of prednisone-equivalent dose (5.59, 2.70 to 11.57, <0.001, vs. ≤10mg/day prednisone), were determined to be associated with a higher risk of COVID-19 related hospitalization. Conversely, 3-dose vaccination significantly reduced the risk of COVID-19-related hospitalization (0.10, 0.02 to 0.40, 0.001, vs. incomplete vaccination) while 2-dose vaccination also provided a trend-towards protective effect against incomplete vaccination (0.43, 0.17 to 1.11, 0.082). Male patients were likely to be at higher risk of COVID-19-related hospitalization (1.98, 0.95 to 4.11, 0.069, vs. female), although not statistically significant (**Figure 2**).

### Clinical factors associated with severe COVID-19 in hospitalized patients

Compared to those with non-severe COVID-19 (n=46), patients with severe COVID-19 (n=19) received a significantly higher dose of prednisone (26.6 vs. 16.3 mg per day, p=0.020) and were more likely to require family oxygen supplement prior to COVID-19 (47.4% vs 21.7%, p=0.077). In addition, patients suffering severe COVID-19 were more likely to receive csDMARDs (52.6% vs. 19.6%) yet were less likely to receive JAKi (31.6% vs. 54.3%) prior to COVID-19 compared to those with non-severe COVID-19 (p=0.056) (**Supplementary Table S1**).

In the secondary analysis (n=65), JAKi pre-exposure was determined to significantly reduce the risk of severe COVID-19 in hospitalized patients (OR=0.16, 95% CI 0.04 to 0.74, p=0.019, vs. csDMARDs), adjusted by age, sex, comorbidities, prednisone dose, and requirement of family oxygen supplement (**Table 3**).

**Table 3:** Clinical factors associated with severe COVID-19 in hospitalized patients determined by multivariable logistic regression.

Candidate predictors for severe COVID-19 (n=65)	OR	95% CI	p value
Immunosuppressant			
Sole csDMARDs	reference		
None	0.11	0.01-1.38	0.087
JAK inhibitor	0.16	0.04-0.74	**0.019**
Rituximab	0.28	0.03-2.36	0.241
Prednisone dose, mg/day	1.04	0.99-1.09	0.069
Family oxygen supplement	2.54	0.61-10.58	0.201
Age, years	1.03	0.95-1.12	0.440
Male	0.66	0.15-2.99	0.593
No. of comorbidity			
None	reference		
One	0.44	0.09-2.10	0.305
Two or more	0.57	0.09-3.53	0.541

COVID-19, Coronavirus Disease 2019; OR, odds ratio; CI, confidence interval; JAK, Janus kinase; csDMARDs, conventional synthetic disease-modifying antirheumatic drugs. P-values<0.05 are highlighted in bold.

### COVID-19-related outcomes depending upon different MSAs

**Figure 3:**
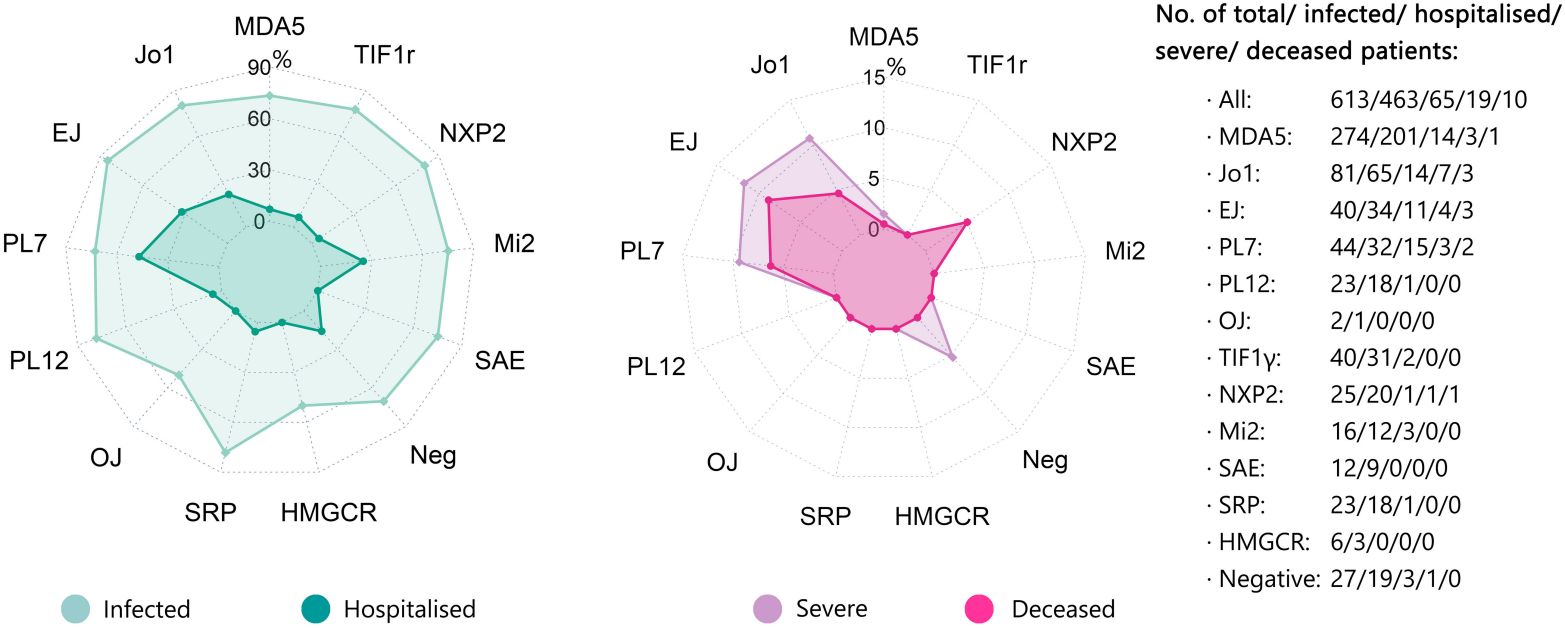
COVID-19 related outcomes (hospitalization, severe, and death) depending upon different MSAs. The rates of SARS-CoV-2 infection, COVID-19-related hospitalization, severe COVID-19, and COVID-19-related death depending upon different MSAs were displayed by radar chart. The corresponding numbers of cases with different MSAs were also shown. COVID-19, Coronavirus Disease 2019; MSA, myositis-specific antibody.

In general, the infection rate of COVID-19 was comparable across different MSAs, mainly varying from 70.4% to 85.0%. However, the rate of hospitalization due to COVID-19 was evidently higher in infected patients with anti-Jo1 antibody (Ab) (21.5%, n=65), anti-EJ Ab (32.4%, n=34), and anti-PL7 Ab (46.9%, n=32) with an appreciable sample size. In addition, the rates of severe COVID-19 and mortality were correspondingly higher in patients with anti-Jo1 Ab (10.8%, 4.6%), anti-EJ Ab (11.8%, 8.8%), and anti-PL7 Ab (9.4%, 6.3%), respectively. The hospitalization rate was 25.0% in patients with anti-Mi2 Ab (n=12) and the mortality was 5.0% in patients with anti-NXP2 Ab (n=20), both slightly above average yet with limited cases. The rates of hospitalization, severe COVID-19, and mortality in patients with anti-MDA5 Ab (n=201) were all below average, i.e., 7.0%, 1.5%, and 0.5%, respectively (**Figure 3**; **Table 4**).

**Table 4:** COVID-19 related outcomes depending upon different MSAs.

MSAs specific-antigen	Infection rate of SARS-CoV-2 (%)	Rate of hospitalization due to COVID-19 (%)	Rate of severe COVID-19 (%)	Overall mortality (%)
MDA5	73.4 (201/274)	7.0 (14/201)	1.5 (3/201)	0.5 (1/201)
Jo1	80.2 (65/81)	**21.5** (14/65)	**10.8** (7/65)	**4.6** (3/65)
EJ	85.0 (34/40)	**32.4** (11/34)	**11.8** (4/34)	**8.8** (3/34)
PL7	72.7 (32/44)	**46.9** (15/32)	**9.4** (3/32)	**6.3** (2/32)
PL12	78.3 (18/23)	5.6 (1/18)	0	0
OJ	50.0 (1/2)	0	0	0
TIF1γ	77.5 (31/40)	6.5 (2/31)	0	0
NXP2	80.0 (20/25)	5.0 (1/20)	5.0 (1/20)	5.0 (1/20)
Mi2	75.0 (12/16)	25.0 (3/12)	0	0
SAE	75.0 (9/12)	0	0	0
SRP	78.3 (18/23)	5.6 (1/18)	0	0
HMGCR	50.0 (3/6)	0	0	0
Negative	70.4 (19/27)	15.8 (3/19)	5.3 (1/19)	0

COVID-19, Coronavirus Disease 2019; MSA, myositis-specific antibody.

The significantly higher rates are highlighted in bold.

### Negative conversion time of antigen test and length of hospitalisation

The negative conversion time of antigen test was available for 388 patients (83.8% of the infected population), with a mean value of 10.5 (4.3) days. It was significantly longer in patients with ASyS (n=128) than those without anti-synthetase antibodies (n=260) (11.3 vs. 10.2 days, p=0.017). Not surprisingly, it was evidently longer in hospitalized patients (n=61) than those not-requiring hospitalization (n=327) (13.8 vs. 9.9 days, p<0.001). However, there was no significant difference between severe (n=15) and non-severe COVID-19 (n=46) in hospitalized patients (14.9 vs. 13.5 days, p=0.290) (**Supplementary Table S2**).

Data for the length of hospitalization were available for 52 patients (80% of hospitalized population), with a mean value of 17.9 (13.2) days. It was comparable between patients with ASyS (n=36) and those without anti-synthetase antibodies (n=16) (18.2 vs. 17.3 days, p=0.821). As expected, severe COVID-19 patients (n=18) stayed significantly longer in the hospital than non-severe patients (n=34) (25.9 vs. 13.7 days, p=0.009) (**Supplementary Table S2**).

### Distinct immunophenotype features of ASyS patients with COVID-19

**Figure 4:**
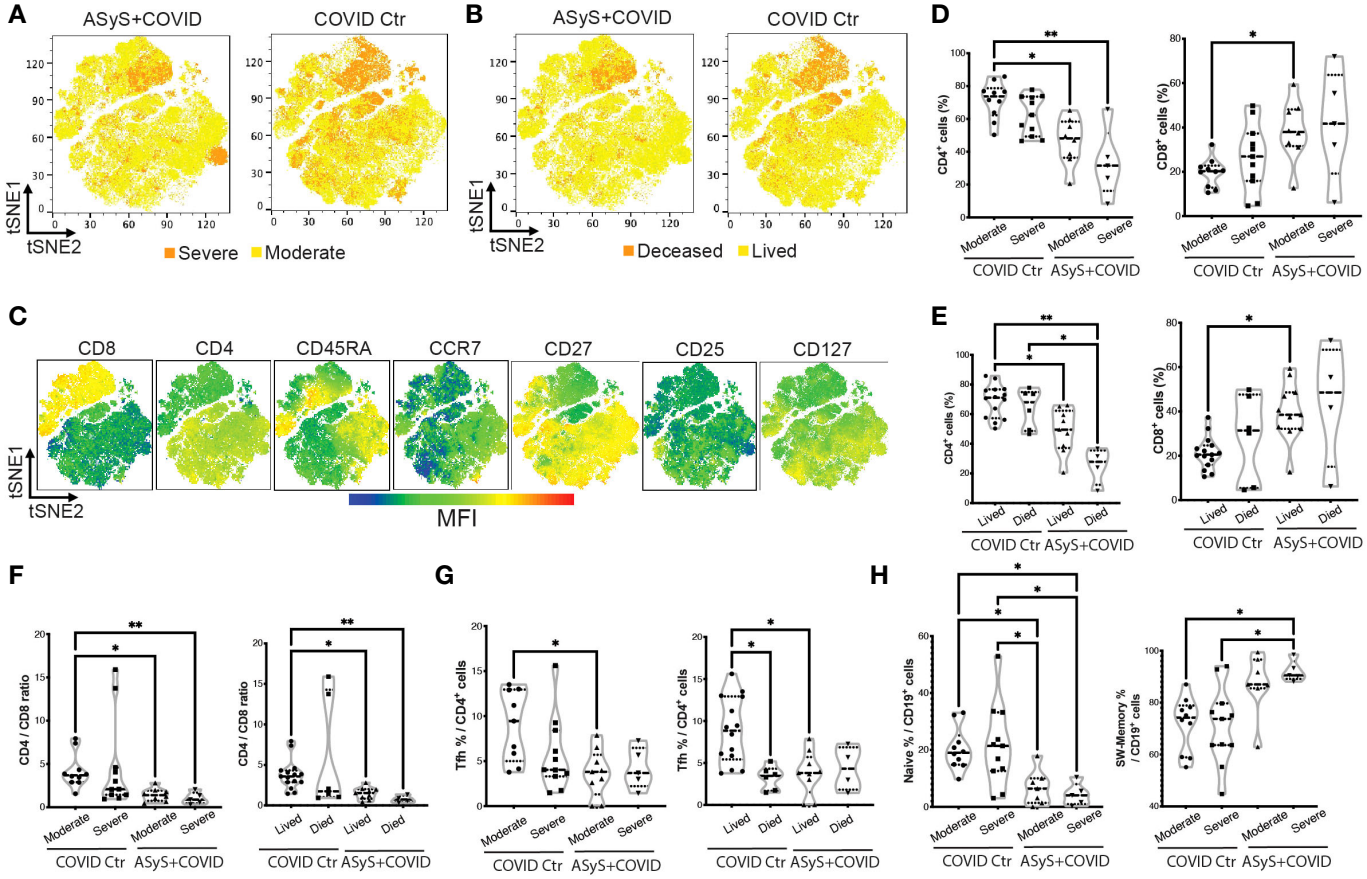
Immune features of hospitalized ASyS patient with COVID-19 by high-dimensional flow cytometry analysis. **(A–C)** tSNE plots of T cells from ASyS patients with COVID-19 (ASyS+COVID) or COVID-19 control (COVID Ctr) colored by **(A)** severity, **(B)** outcome, **(C)** mean fluorescence intensity (MFI) of representative clustering markers. **(D)** Frequency of CD4+ and CD8+ T cells in patients categorized by disease and severity. **(E)** Frequency of CD4+ and CD8+ T cells in patients categorized by disease and outcome. **(F)** CD4/CD8 ratio categorized by disease, severity, and outcome. **(G)** Frequency of Tfh cells in patients categorized by disease, severity, and outcome. **(H)** Frequency of naïve B cells (IgD+CD27-) and class-switched B cells (CD27+IgD-) in patients categorized by disease, severity, and outcome. ASyS, anti-synthetase syndrome; COVID-19, Coronavirus Disease 2019. Kruskal-Wallis test was applied. *p≤0.05, **p≤0.01.

To further address the question of why ASyS+COVID patients were subjected to unfavorable outcomes, a total of 221,696 viable T cells from 14 ASyS+COVID donors and 21 COVID-19 control donors were used for high-dimensional flow cytometry analysis. The composition of cells by donor (**Supplementary Figure S2A**), severity (**Figure 4A**), and clinical outcome (**Figure 4B**) from the ASyS+COVID group and COVID control group (**Supplementary Table S3**) were visualized with tSNE. The expression of clustering markers was normalized and overlaid on tSNE plots (**Figure 4C**; **Supplementary Figure S2B**). Consistent with previous reports, at the time of admission, severe (**Figure 4D**) or fatal (**Figure 4E**) COVID cases showed a trend of decreased CD4+ T cells and increased CD8+ T cells compared to COVID donors with moderate disease and those eventually discharged. This trend is even more remarkable in ASyS patients, particularly in those with severe or fatal COVID-19. ASyS patients who succumbed to COVID had an average of 25.2% CD4+ T cells in their circulating T lymphocytes, which is significantly lower than ASyS patients with moderate COVID (48.6%) and COVID controls (67.0%) (**Figure 4E**). ASyS patients with severe or fatal COVID had an evidently lower CD4/CD8 ratio than COVID controls (**Figure 4F**), indicating a potential role of CD4/CD8 ratio as an identifier for high-risk ASyS patients. To test this idea, we pulled laboratory records of another 36 IIM+COVID patients and divided them into two groups by either positive or negative for anti-synthetase-specific antibodies. A significantly lower CD4/CD8 ratio was found in severe or fatal ASyS+COVID compared to non-ASyS IIM donors with moderate COVID-19 (**Supplementary Figure S2C**), suggesting that the prominent loss of CD4+ T cells and relative expansion of CD8+ T cells is a distinct feature of high-risk ASyS suffered from COVID-19.

As visualized in **Figures 4A–C**, cells from donors with severe or fatal COVID are clustered near the upper right quadrant with low CD45RA, CCR7, CD27, and CD127 expression. For detailed phenotyping, we performed unsupervised clustering to CD8+ and CD4+ T cells respectively using FlowSOM. For CD8+ T cells, 50%-80% of the cells clustered together to form a metacluster 4 (MC4) with Tem features (CD27^-^CD45RA^lo^CCR7^lo^), which was higher in severe or fatal ASyS patients (**Supplementary Figures S2D–F**). For CD4+ T cells (**Supplementary Figures S3A–C**), the largest population showed a naive feature (MC5, CD45RA^+^CCR7^+^) with a moderate level of CXCR3 expression, which was more abundant in moderate cases over severe or fatal cases for both COVID control and ASyS patients. CD4 MC1-4 showed a Tem feature, among which MC1 (CD27^-^Tem) was higher in severe or fatal COVID controls and MC4 (CD25^hi^CD127^lo^ Tregs) was significantly lower in ASyS patients (confirmed by biaxial gating in **Supplementary Figure S3D**). The MC7 (PD-1^+^CXCR5^hi^) Tfh population was significantly lower in ASyS patients (**Figure 4G**; **Supplementary Figure S3C**). However, severe or fatal AsyS cases showed the highest frequency of class-switched memory B cells (CD27^+^IgD^-^) and the lowest frequency of naive B cells (IgD^+^CD27^-^) (**Figure 4H**; **Supplementary Figure S3E**).

## Discussion

In this large cohort comprising 613 Chinese IIM patients, approximately three quarters were infected by SARS-CoV-2 during a short period (within two months). The rates of COVID-19-related hospitalization, severe COVID-19, and mortality were 14.0%, 4.1%, and 2.2%, respectively. As a tertiary referral center, IIM-associated ILD is a predominant feature (~85%) in our cohort due to the high proportion of ASyS and anti-MDA5 positive DM patients. However, only patients with extensive ILD requiring chronic oxygen supplement (~10% prevalence) were at higher risk for COVID-19-related hospitalization. Known risk factors for COVID-19-related hospitalization, such as older age, higher disease activity, and higher GC intake, were consistent with published data (3, 10, 11). In addition, 3-dose inactivated vaccination was found to provide a significant protective effect against hospitalization, which would facilitate vaccination strategy in those countries where mRNA or vector vaccines are not supplied.

It was a novel finding that ASyS is related to unfavorable outcomes of COVID-19. In fact, anti-Jo1 positivity has been reported as a potential risk factor for hospitalization in IIM patients with COVID-19 in a small Hungarian cohort (n=35) (20). Of note, this correlation was reinforced in our study with a ten-fold larger sample size. Patients with anti-EJ Ab and anti-PL7 Ab were also found to be at higher risk of severe COVID-19 and poor outcomes. In comparison, dermatomyositis with anti-MDA5 Ab, a well-known disease linked to progressive ILD, was surprisingly not associated with severe COVID-19 in our cohort. This finding supports that MSAs-guided risk stratification is required for IIM management in the context of COVID-19 infection.

Cumulative evidence has supported the utility of JAKi, including tofacitinib and baricitinib, in dermatomyositis patients (21–23), especially among anti-MDA5 positive DM (24). An intriguing finding of our study is that JAKi pre-exposure might be beneficial in terms of the prognosis of hospitalized IIM patients with COVID-19. Indeed, JAKi has shown strong evidence to improve survival in the general population hospitalized with COVID-19, such as baricitinib in the the COV-BARRIER and RECOVERY trials (25–27), making it recommended for severe and critical COVID-19 patients requiring oxygen supplement by WHO and NIH guidelines (28). It is noteworthy that our data should not be extrapolated for the overuse of JAK inhibitors among IIM patients. More data are required before a solid conclusion can be made.

Many studies in the literature have described the immunology of SARS-CoV-2 infection. However, few studies touched on the impact of COVID-19 with pre-existing IIM (29, 30). In this study, a prominent reduction of CD4+ T cells was found in severe ASyS+COVID cases and a lower CD4/CD8 ratio could serve as a convenient biomarker to identify high-risk ASyS patients with COVID-19. Moreover, altered frequencies of CD4+ and CD8+ T cell subsets including expansion of CD27^-^ Tem, reduction of Tfh and Treg, increase of class-switched memory B cells, and loss of naive B cells were features of ASyS with severe or fatal COVID-19. In the general population, compared to non-severe cases, severe infection was associated with lower lymphocyte counts, increased CD4+ and CD8+ Tem, and relative preservation of Treg over Th1/Th17 cells (31). Interestingly, a pattern of ‘coexisting suppression and activation’ may explain the loss of CD4+ T cells and inversed CD4/CD8 ratio during COVID-19, and the further exaggeration of this trend in ASyS patients may indicate a shared pathology involving dysregulated lymphocyte proliferation, apoptosis, or tissue chemotaxis of certain pathological lymphocyte subsets (32).

Recently, an increased incidence of IIMs was reported during the COVID-19 pandemic (33, 34). It has been postulated that an overactive innate immune response to SARS-CoV-2 might contribute to the development and deterioration of autoimmune diseases. In addition, the target antigen in ASyS, i.e., Aminoacyl tRNA synthetases (ARS), might be multifacetly involved in the immune response to virus infection (35). In light of the report that most SARS-CoV-2-infected cells expressed CD27 (36), the loss of CD27 on CD4+ and CD8+ T cells seen in ASyS patients with severe COVID-19 may indicate a lack of anti-viral immunity. Considering the paradoxical reduction of Tfh and increase of class-switched memory B cells, the mechanism behind uncontrolled infection and poor outcome in ASyS may involve a futile immune response against the virus. This hypothesis might be supported by our data which indicated that the negative conversion time of antigen test was longer in patients with ASyS compared to other IIMs. SARS-CoV-2 infection downregulated several tRNA-synthetases’ expression in serum and tissue (37), while a dramatic reduction of circulating his-tRNA synthetase (HARS) was also found in anti-Jo1 positive ASyS and HARS administration ameliorated immune over-activation (38). Thus, reduced tRNA synthetase along with immunological disturbance may contribute to the severity of COVID in ASyS, and investigating the role of certain tRNA synthetases may identify therapeutic targets to protect vulnerable ASyS patients.

This study has several strengths. First, the study was based on a large and high-quality IIM cohort. Second, massive SARS-CoV-2 Omicron variant infections during a short period of time provided a neat cause-effect model and many confounding issues such as the evolving of viral variants and changing of treatment protocols over time could be largely eliminated. The robustness of our data was evident by the reproducibility of many well-known risk factors in this study.

This study also has some limitations. First, as a rheumatology referral center, IIM patients with more visceral organ involvements, e.g., ASyS and anti-MDA5 positive dermatomyositis complicated with ILD, were largely enriched in our cohort. On the other hand, this referral bias provided a valuable opportunity to investigate these vulnerable patients. Second, few patients in our cohort received timely outpatient anti-viral therapy from the primary care system, e.g., Paxlovid within 5 days since COVID-19 onset (39), which hindered this important treatment factor from being incorporated in the core analysis. Third, some rare MSAs were not included in the EUROLINE assay, such as anti-HA, anti-Zo, and anti-KS (anti-synthetase) Abs, although the impact should be predictably minimal.

In conclusion, COVID-19-related outcomes were reported in this large IIM cohort. ASyS, requiring family oxygen supplement prior to infection, along with older age, higher IIM disease activity, and high-dose GC exposure, were identified as risk factors for COVID-19-related hospitalization. Three-dose inactivated vaccination significantly reduced the risk of hospitalization. JAKi pre-exposure might have a protective effect in IIM against evolution to severe COVID-19. Our findings highlighted special caution for patients with ASyS suffering from COVID-19, especially for those with a low CD4/CD8 ratio, and further mechanistic study may provide insights to better understand the immunopathogenesis of this unique scenario.

## Data availability statement

The raw data supporting the conclusions of this article will be made available by the authors on reasonable request to the corresponding authors, without undue reservation.

## Ethics statement

The studies involving humans were approved by Ethics Committee of Renji Hospital (identification no. 2013-126, RA-2023-101). The studies were conducted in accordance with the local legislation and institutional requirements. The participants provided their written informed consent to participate in this study.

## Author contributions

WW: Conceptualization, Data curation, Formal analysis, Investigation, Methodology, Project administration, Validation, Writing – original draft. RW: Conceptualization, Data curation, Formal analysis, Funding acquisition, Investigation, Methodology, Writing – original draft. CX: Writing – review & editing, Conceptualization, Data curation, Formal analysis, Validation. YC: Data curation, Resources, Supervision, Validation, Writing – review & editing. XT: Investigation, Methodology, Project administration, Resources, Supervision, Writing – review & editing. SS: Data curation, Validation, Writing – review & editing. WX: Data curation, Validation, Writing – review & editing. YF: Data curation, Validation, Writing – review & editing. YM: Data curation, Validation, Writing – review & editing. AX: Data curation, Validation, Writing – review & editing. XL: Data curation, Validation, Writing – review & editing. YY: Data curation, Validation, Writing – review & editing. JL: Conceptualization, Data curation, Methodology, Validation, Writing – review & editing. CZ: Data curation, Resources, Validation, Writing – review & editing. NS: Conceptualization, Methodology, Resources, Supervision, Writing – review & editing. XW: Conceptualization, Data curation, Supervision, Writing – review & editing. SY: Conceptualization, Data curation, Formal analysis, Investigation, Methodology, Project administration, Resources, Supervision, Writing – review & editing. QF: Conceptualization, Data curation, Formal analysis, Funding acquisition, Investigation, Methodology, Project administration, Resources, Supervision, Writing – review & editing.
