# Supplementary material for: Anti-synthetase syndrome is associated with a higher risk of hospitalization among patients with idiopathic inflammatory myopathy and COVID-19
Source: Front Immunol. 2024 Mar 4;15:1295472. doi: 10.3389/fimmu.2024.1295472 (PMC10944926; doi:10.3389/fimmu.2024.1295472)
Supplement: Supplementary file 1 [file DataSheet_1.docx]

**Table S1** Univariable comparisons of clinical features prior to COVID-19 and outcomes between severe and non-severe COVID-19 in hospitalized patients

|  | Overall  (n=65) | Non-severe  (n=46) | Severe  (n=19) | p value |
| --- | --- | --- | --- | --- |
| Age, years | 59.2 (9.8) | 58.5 (9.4) | 60.7 (10.7) | 0.421 |
| Sex |  |  |  | 1.000 |
| Female | 41 (63.1%) | 29 (63.0%) | 12 (63.2%) |  |
| Male | 24 (36.9%) | 17 (37.0%) | 7 (36.8%) |  |
| Disease duration, years | 3.7 (3.8) | 4.2 (4.3) | 2.6 (1.8) | 0.130 |
| Interstitial lung disease | 61 (93.8%) | 42 (91.3%) | 19 (100.0%) | 0.448 |
| Family oxygen supplement | 19 (29.2%) | 10 (21.7%) | 9 (47.4%) | **0.077** |
| MSAs-gauged IIM subtypes |  |  |  | 0.678 |
| Anti-MDA5 DM | 14 (21.5%) | 11 (23.9%) | 3 (15.8%) |  |
| ASyS | 41 (63.1%) | 27 (58.7%) | 14 (73.7%) |  |
| IMNM | 1 (1.5%) | 1 (2.2%) | 0 (0.0%) |  |
| Other DM | 9 (13.8%) | 7 (15.2%) | 2 (10.5%) |  |
| Vaccination |  |  |  | 0.372 |
| Incomplete (0-1 dose) | 54 (83.1%) | 40 (87.0%) | 14 (73.7%) |  |
| Full (2 doses) | 8 (12.3%) | 4 (8.7%) | 4 (21.1%) |  |
| Booster (3 doses or more) | 3 (4.6%) | 2 (4.3%) | 1 (5.3%) |  |
| IIM disease activity (PtGA) |  |  |  | 0.348 |
| Remission | 9 (13.8%) | 8 (17.4%) | 1 (5.3%) |  |
| Low | 25 (38.5%) | 17 (37.0%) | 8 (42.1%) |  |
| Moderate | 23 (35.4%) | 17 (37.0%) | 6 (31.6%) |  |
| High | 8 (12.3%) | 4 (8.7%) | 4 (21.1%) |  |
| Prednisone, mg/day | 19.3 (16.4) | 16.3 (10.8) | 26.6 (24.2) | **0.020** |
| Prednisone dose>10mg/day | 43 (66.2%) | 30 (65.2%) | 13 (68.4%) | 1.000 |
| Withdrawn of GC | 1 (1.5%) | 1 (2.2%) | 0 (0.0%) | 1.000 |
| No. of immunosuppressant |  |  |  | 0.518 |
| None | 8 (12.3%) | 7 (15.2%) | 1 (5.3%) |  |
| Mono | 43 (66.2%) | 29 (63.0%) | 14 (73.7%) |  |
| Combined | 14 (21.5%) | 10 (21.7%) | 4 (21.1%) |  |
| Type of immunosuppressant |  |  |  | **0.056** |
| None | 8 (12.3%) | 7 (15.2%) | 1 (5.3%) |  |
| JAK inhibitor | 31 (47.7%) | 25 (54.3%) | 6 (31.6%) |  |
| sole csDMARDs | 19 (29.2%) | 9 (19.6%) | 10 (52.6%) |  |
| Rituximab | 7 (10.8%) | 5 (10.9%) | 2 (10.5%) |  |
| No. of comorbidity |  |  |  | 0.628 |
| None | 25 (38.5%) | 16 (34.8%) | 9 (47.4%) |  |
| One | 23 (35.4%) | 17 (37.0%) | 6 (31.6%) |  |
| Two or more | 17 (26.2%) | 13 (28.3%) | 4 (21.1%) |  |
| Smoker |  |  |  | 0.877 |
| Never | 53 (81.5%) | 37 (80.4%) | 16 (84.2%) |  |
| Ever | 9 (13.8%) | 7 (15.2%) | 2 (10.5%) |  |
| Current | 3 (4.6%) | 2 (4.3%) | 1 (5.3%) |  |
| BMI, kg/m^2^ | 24.3 (3.1) | 24.5 (3.1) | 23.6 (3.0) | 0.276 |
| BMI>28 | 8 (12.3%) | 8 (17.4%) | 0 (0.0%) | 0.093 |
| Death | 10 (15.4%) | 0 (0.0%) | 10 (52.6%) | **<0.001** |

Data are presented as mean (SD), or n (%).

COVID-19=Coronavirus Disease 2019. IIM=idiopathic inflammatory myopathy. MSA=myositis-specific antibody. MDA5=melanoma differentiation-associated gene 5. DM=dermatomyositis. ASyS=anti-synthetase syndrome. IMNM=immune-mediated necrotizing myopathy. PtGA=patient’s global assessment. GC=glucocorticoid. JAK=Janus kinase. csDMARDs=conventional synthetic disease-modifying antirheumatic drugs. BMI=Body Mass Index.

**Table S2** Negative conversion time of antigen test and length of hospitalization stratified by MSAs, hospitalization, and severity of COVID-19

|  |  | Negative conversion time of antigen test / days | p value |
| --- | --- | --- | --- |
| MSAs-gauged IIM subtypes | ASyS, n=128 | 11.3 (4.4) | Ref |
|  | Non-ASyS, n=260 | 10.2 (4.1) | **0.017** |
|  | Other DM, n=78 | 10.1 (4.0) | 0.055 |
|  | Anti-MDA5 DM, n=168 | 10.3 (4.3) | 0.052 |
|  | IMNM, n=14 | 9.5 (3.1) | 0.144 |
| Hospitalization | Yes, n=61 | 13.8 (4.4) | **<0.001** |
|  | No, n=327 | 9.9 (4.0) |  |
| Severe COVID-19 in hospitalized patients | Yes, n=15 | 14.9 (5.2) | 0.290 |
|  | No, n=46 | 13.5 (4.1) |  |
|  |  | **Length of hospitalization / days** | **p value** |
| MSAs-gauged IIM subtypes | ASyS, n=36 | 18.2 (14.2) | 0.821 |
|  | Non-ASyS, n=16 | 17.3 (10.8) |  |
| Severe COVID-19 | Yes, n=18 | 25.9 (17.0) | **0.009** |
|  | No, n=34 | 13.7 (8.1) |  |

Data are presented as mean (SD).

COVID-19=Coronavirus Disease 2019. IIM=idiopathic inflammatory myopathy. MSA=myositis-specific antibody. MDA5=melanoma differentiation-associated gene 5. DM=dermatomyositis. ASyS=anti-synthetase syndrome. IMNM=immune-mediated necrotizing myopathy.

**Table S3** Clinical factors of patients with ASyS+COVID and COVID-19 control used in immunophenotyping study

|  | ASyS+COVID | | COVID-19 control | |
| --- | --- | --- | --- | --- |
|  | Moderate (n=10) | Severe (n=5) | Moderate (n=10) | Severe (n=11) |
| Age, years | 64.4 (10.2) | 57.0 (6.3) | 73.4 (8.4) | 74.4 (9.1) |
| Sex |  |  |  |  |
| Female | 7 (70%) | 4 (80%) | 7 (70%) | 3 (27%) |
| Male | 3 (30%) | 1 (20%) | 3 (30%) | 8 (73%) |
| Disease duration of IIM, years | 2.6 (2.4) | 2.5 (1.5) | / | / |
| Interstitial lung disease | 10 (100%) | 5 (100%) | / | / |
| Previous use of glucocorticoids | 10 (100%) | 5 (100%) | / | / |
| Previous use of immunosuppressants | 9 (90%) | 4 (80%) | / | / |
| BMI, kg/m^2^ | 24.6 (4.3) | 24.8 (2.8) | 24.6 (2.6) | 23.4 (4.4) |
| Comorbidity |  |  |  |  |
| Hypertension | 3 (30%) | 1 (20%) | 4 (40%) | 4 (36%) |
| Coronary heart disease | 1 (10%) | 0 (0%) | 0 (0%) | 4 (36%) |
| Diabetes | 3 (30%) | 1 (20%) | 3 (30%) | 1 (9%) |
| Malignancy | 0 (0%) | 0 (0%) | 2 (20%) | 2 (18%) |
| COPD | 0 (0%) | 0 (0%) | 1 (10%) | 0 (0%) |
| Chronic renal disease | 0 (0%) | 0 (0%) | 1 (10%) | 1 (9%) |
| Stroke | 1 (10%) | 0 (0%) | 1 (10%) | 2 (18%) |
| Death | 0 (0%) | 4 (80%) | 0 (0%) | 6 (55%) |

Data are presented as mean (SD), or n (%).

ASyS=anti-synthetase syndrome. COVID-19=Coronavirus Disease 2019. BMI=Body Mass Index. COPD=chronic obstructive pulmonary disease.

**Figure S1** Gating strategy used in flow cytometry manual gating


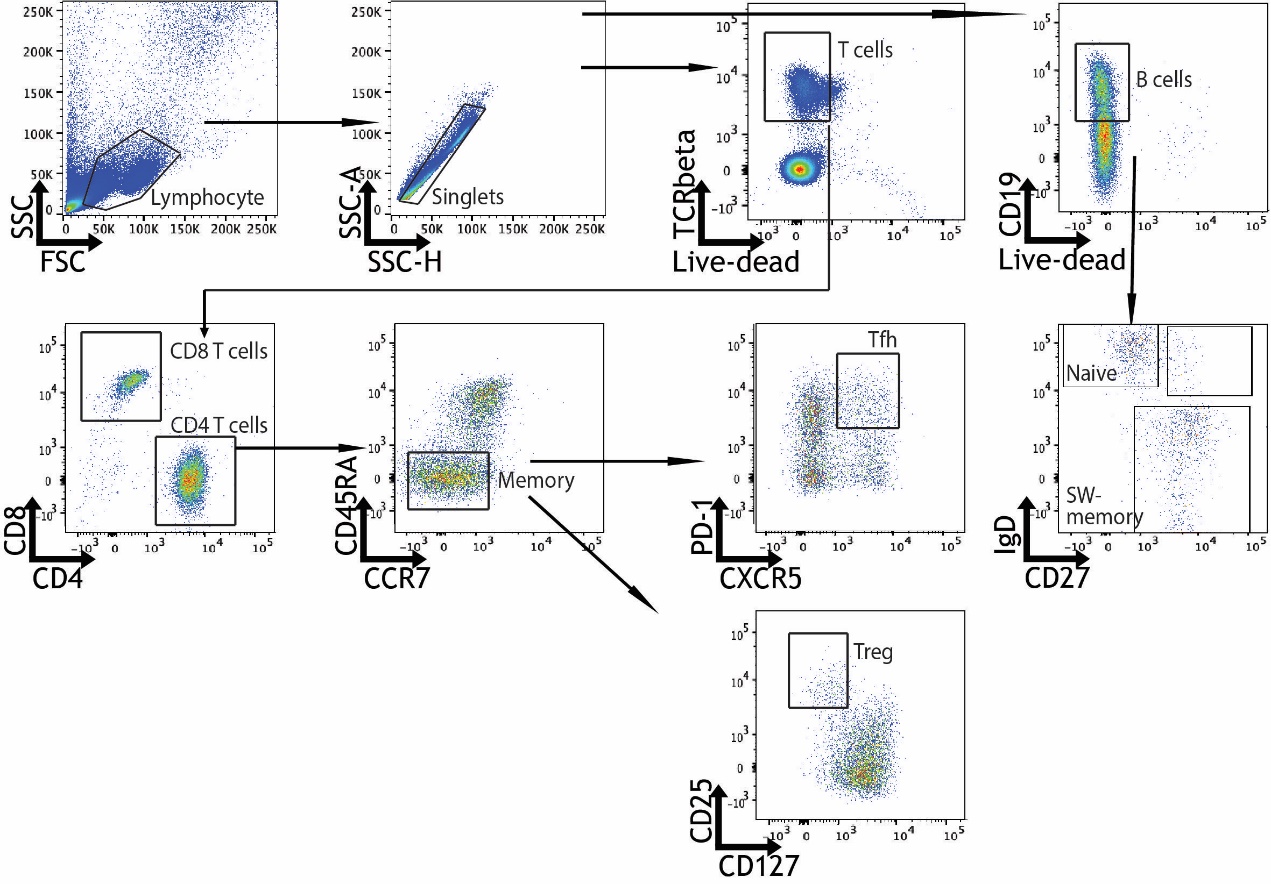


Live TCRβ+ T cells or CD19+ B cells were gated from singlet lymphocyte population. From T cells, CD8+ and CD4+ cells were gated and memory CD4+ T cells were further gated to define PD-1hi CXCR5- Tfh cells and CD25hiCD127- Treg cells. From B cells, naive or class-switched memory B cells were gated based on IgD and CD27 expression.

**Figure S2** Additional immune features and high-dimensional analysis of CD8+ T cells


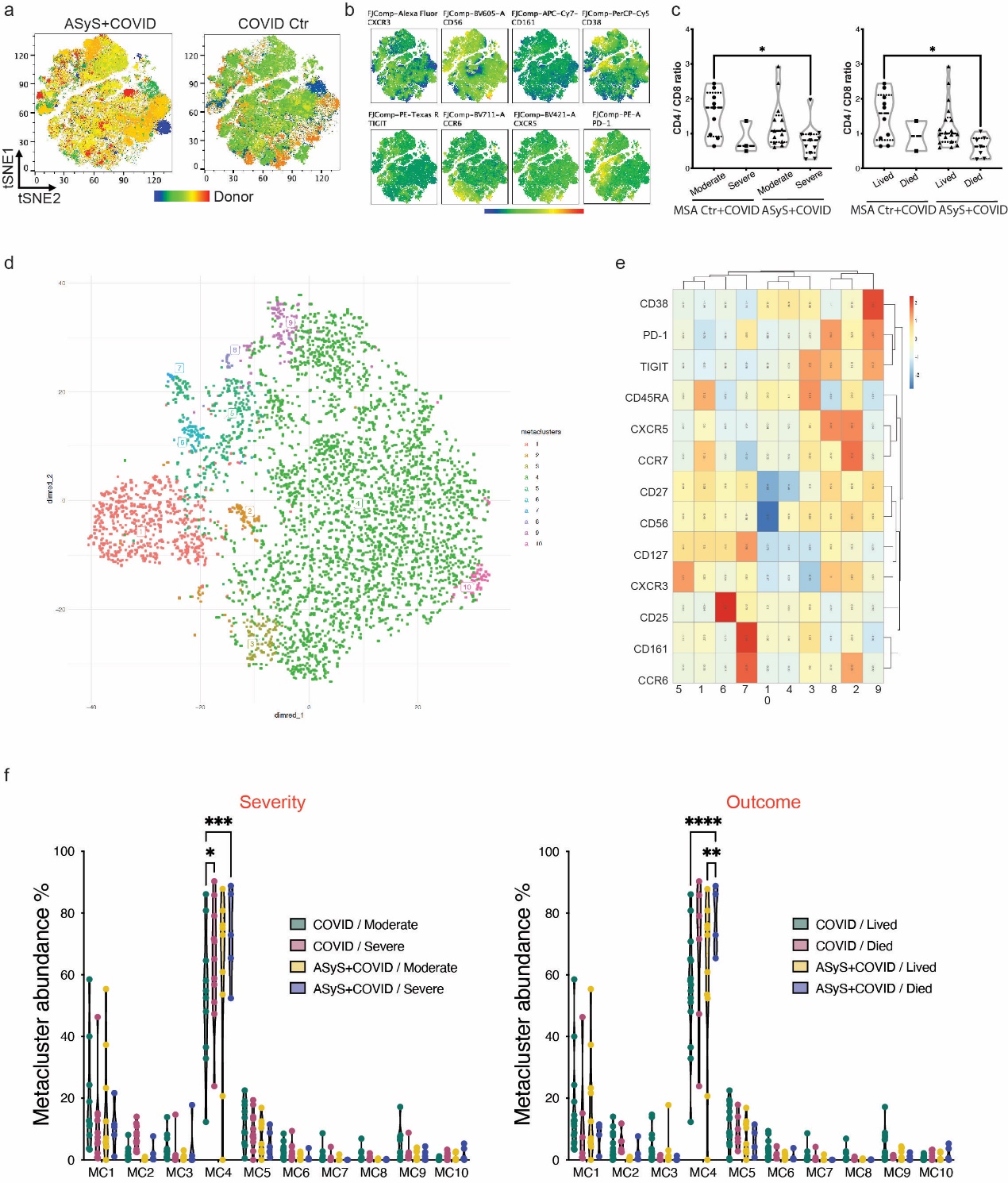


a). tSNE plots of T cells from ASyS patients or COVID control colored by donor. b). tSNE plots of T cells from ASyS patients or COVID control colored MFI of additional clustering markers. c). CD4/CD8 ratio from clinical laboratory records of COVID patients with myositis-specific autoantibodies. MSA: COVID cases with non-ASyS MSA. d). tSNE plots showing 10 metaclusters of CD8+ T cells from ASyS+COVID and COVID control, colored by metacluster ID. e). Heatmap showing MFI of clustering markers in each metacluster. f). Abundance of CD8 metaclusters in patients categorised by severity (left) or outcome (right).

ASyS=anti-synthetase syndrome. COVID-19=Coronavirus Disease 2019. MFI=mean fluorescence intensity. MSA=myositis-specific antibody.

**Figure S3** Additional immune features and high-dimensional analysis of CD4+ T cells and B cells


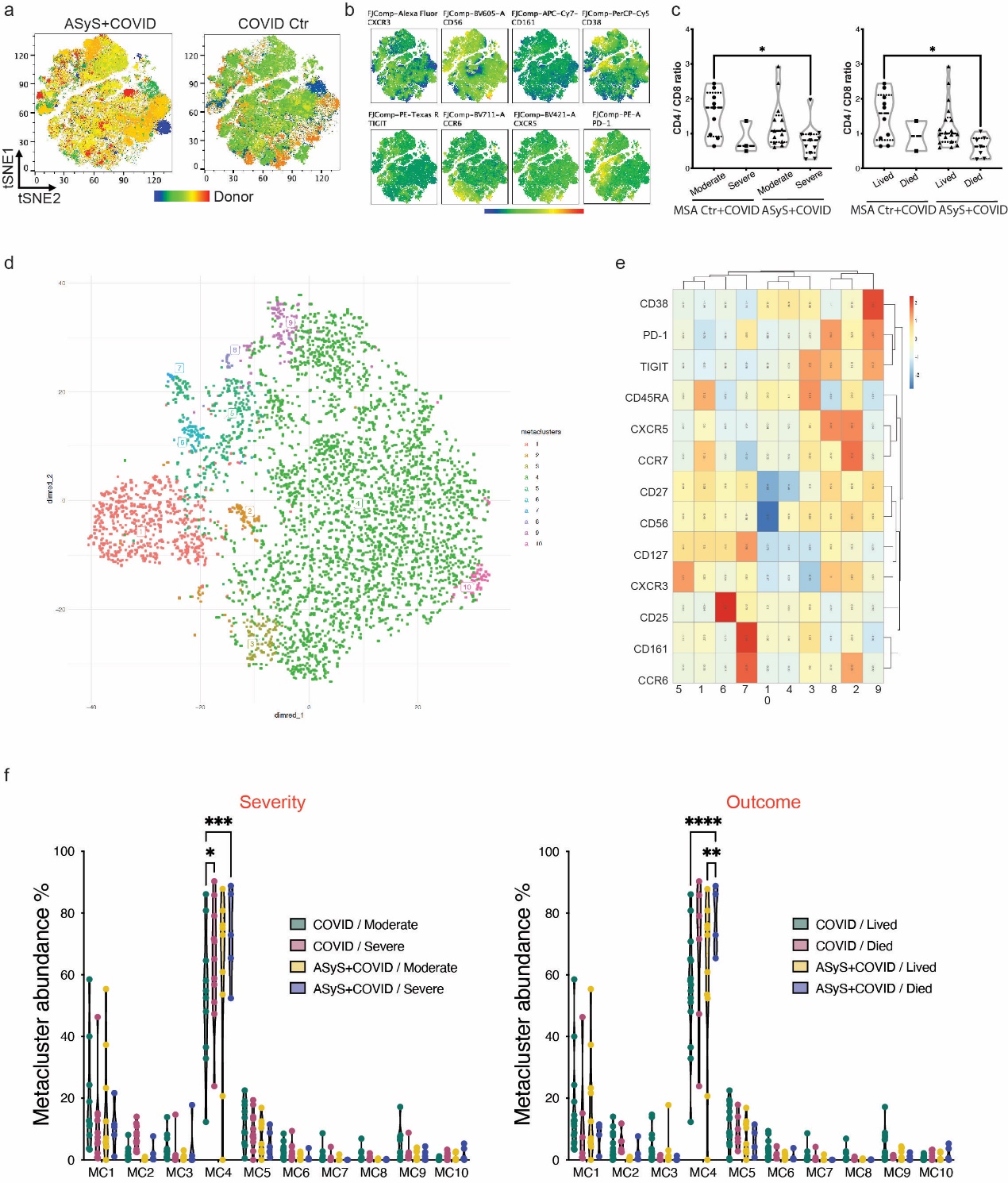


a). tSNE plots showing 10 metaclusters of CD4+ T cells from ASyS+COVID and COVID control, colored by metacluster ID. b). Heatmap showing MFI of clustering markers in each metacluster. c). Abundance of CD4+ metaclusters in patients categorised by severity (left) or outcome (right). d). Frequency of Treg cells in patients categorised by disease, severity and outcome. e). Frequency of naive B cells and class-switched B cells in patients categorised by disease, severity and outcome.

ASyS=anti-synthetase syndrome. COVID-19=Coronavirus Disease 2019. MFI=mean fluorescence intensity.
